# Cellular and Molecular Pathways of COVID-19 and Potential Points of Therapeutic Intervention

**DOI:** 10.3389/fphar.2020.01169

**Published:** 2020-07-29

**Authors:** John P. Hussman

**Affiliations:** Hussman Foundation, Ellicott City, MD, United States

**Keywords:** COVID-19, immunity, therapeutics, signal transduction, cytokines

## Abstract

With the objective of linking early findings relating to the novel SARS-CoV-2 coronavirus with potentially informative findings from prior research literature and to promote investigation toward therapeutic response, a coherent cellular and molecular pathway is proposed for COVID-19. The pathway is consistent with a broad range of observed clinical features and biological markers and captures key mediators of pathophysiology. In this proposed pathway, membrane fusion and cytoplasmic entry of SARS-CoV-2 virus *via* ACE2 and TMPRSS2-expressing respiratory epithelial cells, including pulmonary type-II pneumocytes, provoke an initial immune response featuring inflammatory cytokine production coupled with a weak interferon response, particularly in IFN-λ–dependent epithelial defense. Differentiation of non-classic pathogenic T-cells and pro-inflammatory intermediate monocytes contributes to a skewed inflammatory profile, mediated by membrane-bound immune receptor subtypes (e.g., Fc*γ*RIIA) and downstream signaling pathways (e.g., NF-κB p65 and p38 MAPK), followed by chemotactic infiltration of monocyte-derived macrophages and neutrophils into lung tissue. Endothelial barrier degradation and capillary leakage contribute to alveolar cell damage. Inflammatory cytokine release, delayed neutrophil apoptosis, and NETosis contribute to pulmonary thrombosis and cytokine storm. These mechanisms are concordant with observed clinical markers in COVID-19, including high expression of inflammatory cytokines on the TNF-α/IL-6 axis, elevated neutrophil-to-lymphocyte ratio (NLR), diffuse alveolar damage *via* cell apoptosis in respiratory epithelia and vascular endothelia, elevated lactate dehydrogenase (LDH) and CRP, high production of neutrophil extracellular traps (NETs), depressed platelet count, and thrombosis. Although certain elements are likely to be revised as new findings emerge, the proposed pathway suggests multiple points of investigation for potential therapeutic interventions. Initial candidate interventions include prophylaxis to augment epithelial defense (e.g., AT1 receptor blockade, type III and type I interferons, melatonin, calcitriol, camostat, and lopinavir) and to reduce viral load (e.g., remdesivir, ivermectin, emetine, Abelson kinase inhibitors, dopamine D2 antagonists, and selective estrogen receptor modulators). Additional interventions focus on tempering inflammatory signaling and injury (e.g., dexamethasone, doxycycline, Ang1-7, estradiol, alpha blockers, and DHA/EPA, pasireotide), as well as inhibitors targeted toward molecular mediators of the maladaptive COVID-19 immune response (e.g., IL-6, TNF-α, IL-17, JAK, and CDK9).

## Introduction

COVID-19 is a severe acute respiratory disease caused by the novel coronavirus SARS-CoV-2, which emerged in Wuhan, China in late 2019, quickly becoming a global pandemic, with over 10 million reported cases and 500,000 fatalities attributed to the disease through June 2020. Much of the response to the novel coronavirus has relied, by necessity, on a broad range of early reports relating to clinical features, biological markers, and candidate therapeutics. At the same time, many characteristics of the SARS-CoV-2 coronavirus and the acute respiratory distress produced by severe cases of COVID-19 infection mirror those observed in earlier coronavirus outbreaks, including SARS (severe acute respiratory syndrome, caused by SARS-CoV) and MERS (Middle-East respiratory syndrome, caused by MERS-CoV). Other conditions with informative overlap include ARDS (acute respiratory distress syndrome, resulting from pulmonary edema) and dengue hemorrhagic fever (DHF), which features severe and often fatal secondary immunopathology following dengue virus infection (Kurane, 2007) involving rapidly elevated cytokine expression, pulmonary edema, and acute respiratory failure.

The SARS-CoV-2 epidemic has emerged in the context of a rich existing literature detailing aspects of cellular and molecular pathways affected by prior CoV serotypes and related conditions. Much of the emerging literature specific to SARS-CoV-2 not only is strongly consistent with these findings but also features informative differences, particularly in lung tissue (e.g., weaker type III and type I interferon response, suppressed epithelial defense, and elevated pulmonary infectivity).

With the objective of linking early findings relating to the novel SARS-CoV-2 coronavirus with potentially informative findings from prior research literature and to promote investigation toward therapeutic response, a coherent cellular and molecular pathway is proposed for COVID-19. The pathway is consistent with a broad range of observed clinical features and biological markers and captures key mediators of pathophysiology.

In this proposed pathway, membrane fusion and cytoplasmic entry of SARS-CoV-2 virus *via* ACE2 and TMPRSS2-expressing respiratory epithelial cells, including pulmonary type-II pneumocytes, provokes an initial immune response featuring inflammatory cytokine production coupled with a weak interferon response, particularly in IFN-λ–dependent epithelial defense. Differentiation of non-classic pathogenic T-cells and pro-inflammatory intermediate monocytes contributes to a skewed inflammatory profile, mediated by membrane-bound immune receptor subtypes (e.g., Fc*γ*RIIA) and downstream signaling pathways (e.g., NF-κB p65 and p38 MAPK), followed by chemotactic infiltration of monocyte-derived macrophages and neutrophils into lung tissue. Endothelial barrier degradation and capillary leakage contribute to alveolar cell damage. Inflammatory cytokine release, delayed neutrophil apoptosis, and NETosis contribute to pulmonary thrombosis and cytokine storm. These mechanisms are concordant with observed clinical markers in COVID-19, including high expression of inflammatory cytokines on the TNF-α/IL-6 axis, elevated neutrophil-to-lymphocyte ratio (NLR), diffuse alveolar damage *via* cell apoptosis in respiratory epithelia and vascular endothelia, elevated lactate dehydrogenase (LDH) and C-reactive protein (CRP), high production of neutrophil extracellular traps (NETs), depressed platelet count, and thrombosis.

Although certain elements are likely to be revised as new findings emerge, the proposed pathway suggests multiple points of investigation for potential therapeutic interventions. These include prophylaxis to augment epithelial defense, reduce viral load, and temper inflammatory injury, as well as therapeutics targeted toward molecular mediators of the COVID-19 immune response.

## Clinical Features

Among patients with COVID-19 infection, cellular biomarkers in severe cases include elevated leukocyte and neutrophil counts, along with suppressed lymphocyte count, resulting in a significantly higher NLR ratio relative to non-severe cases (Huang C. et al., 2020; Qin et al., 2020). In a meta-analysis of nine studies including 1779 patients, 399 with severe disease, low platelet count was significantly associated with disease severity and mortality. Platelet count (thrombocytopenia) below the locally defined reference range is associated with a five-fold increase in the risk of severe disease (Lippi et al., 2020).

Molecular biomarkers of severe disease include elevated procalcitonin, serum ferritin, D-dimer, C-reactive protein (CRP), and inflammatory cytokines including IL-6, IL-2R, IL-7, IL-8/CXCL8, IP10, MCP-1/CCL2, MIP1A/CCL3, GM-CSF, and TNF-α, as well as IL-10 (Huang C. et al., 2020; Qin et al., 2020). However, the level of IL-10, a negative regulator of immune response, is reported to vary with COVID-19 severity and progression, with lower initial levels and subsequent decline associated with milder cases and possibly more successful viral clearance (Ouyang et al., 2020). Fast respiratory rate and elevated levels of lactate dehydrogenase (LDH), a marker of cell death, also predict severity (Huang H. et al., 2020).

Elevated inflammatory markers including IL-6, CRP, procalcitonin (PCT), and erythrocyte sedimentation rate (ESR) are observed in fatal cases (Zeng et al., 2020). Fatal acute lung injury is associated with T-lymphocyte dysregulation and cytokine-driven inflammation (Qin et al., 2020), with diffuse pulmonary thrombosis and damage to endothelial cells (Poor et al., 2020).

In examination of postmortem tissue from all major organs of COVID-19 subjects, the primary finding is diffuse alveolar damage (DAD), featuring marked infection and viral burden in type II pneumocytes, along with pulmonary edema (Bradley et al., 2020; Carsana et al., 2020). CT examination is reported to have high diagnostic value, with multiple ground glass opacities being a prominent feature of disease progression (Li and Xia, 2020).

COVID-19 features infiltration of macrophages into lung tissue, with apoptosis of epithelial cells and pneumocytes. Infiltration of macrophages into alveolar cavities may be induced by MCP-1, with TGF-β1 and TNF-α contributing to proliferation and amplified cytokine production (He et al., 2006). Markers of infiltration include the neutrophil chemokine receptor CXCR2, along with monocyte chemotactic protein MCP-1/CCL2 and its receptor CCR2. Genes upregulated in severe and critically ill patients are enriched with members belonging to the NF-κB pathway (Hadjadj et al., 2020). Increased expression of TGF-beta in COVID-19 patients may promote fibroblast proliferation and contribute to pulmonary fibrosis (Xiong et al., 2020).

Several comorbid conditions are cited as risk-factors for progression and case fatality, including age, diabetes, vascular disease, cardiac dysfunction, hypertension, and cancer (Wu and McGoogan, 2020). Fever is the most common initial symptom, followed by cough, with maximum body temperature at admission, respiratory rate, CRP, and albumin significantly associated with progression in severity (Liu W. et al., 2020). Gastrointestinal symptoms are also reported but with lower frequency than in SARS or MERS (Ge et al., 2020).

The conditions associated with severe COVID-19 are not accurately described as “compromised immunity.” Among 5700 hospitalized patients in the New York area with confirmed disease, the most frequent comorbidities reported were hypertension (56.6%), obesity (41.7%), diabetes (33.8%), and coronary artery disease (11.1%) (Richardson et al., 2020), all of which may be better described as conditions featuring predisposition to inflammation. Indeed, several key inflammatory cytokines associated with hypertension (TNF-α, MCP-1, and IL-6) (De Miguel et al., 2015) overlap those elevated in COVID-19.

## ACE2-Mediated Viral Entry and Priming of Inflammatory Response

Like the SARS coronavirus, the novel SARS-CoV-2 virus uses membrane-bound ACE2 to gain access to cells. ACE2 functions as an enzyme within the renin-angiotensin system (RAS), contributing to the regulation of blood pressure, fluid balance, and vasoconstriction. Angiotensin I (Ang I) generated by renin cleavage is converted by angiotensin-converting enzyme ACE to produce Ang II, which in turn activates AT1R receptors, contributing to increased blood pressure, vasoconstriction, oxidative stress, and pro-inflammatory signaling. The ACE2 enzyme has high affinity for Ang II, producing Ang(1-7). ACE2 thereby antagonizes the effects of Ang II and exerts a protective effect in conditions such as diabetes, hypertension, and cardiovascular disease (Cheng et al., 2020). Notably, elevated levels of Ang II are observed in ACE/ARB naïve COVID-19 cases, and high levels are associated with increased severity (Liu N. et al., 2020).

Initial genetic evidence of ACE2-mediated entry by SARS-CoV demonstrated that injection of spike protein in mice contributed to acute lung failure in mice and down-regulation of ACE2 expression. Inhibition of AT1R reduced lung pathology by blocking the effect of Ang II (Kuba et al., 2005). Notably, ACE2 is abundantly expressed on lung alveolar cells and enterocytes of the small intestine and is also present in vascular endothelia (Hamming et al., 2004), consistent with initial presentation of symptoms and sites of subsequent tissue damage.

SARS-CoV-2 viral entry is also dependent on priming of the viral S protein by the serine protease TMPRSS2, which may be partially blocked in some cell types by the serine protease inhibitor camostat mesilate. Full blockade was reported when camostat inhibition of TMPRSS2 was combined with an inhibitor of endosomal cysteine proteases cathepsin B/L (Hoffmann et al., 2020).

Despite exploitation of RAS by SARS-CoV-2, clinical evidence does not support the discontinuation of ACE-inhibitors or AT1R blockers (ARBs) as a strategy to limit infection, particularly as both types of inhibitors act to reduce the hypertensive and pro-inflammatory effects of Ang II. In SARS-CoV-2 infection, virus-induced ACE2 downregulation would be expected to lead to reduced production of Ang(1-7) and accumulation of Ang II, contributing to pulmonary edema and inflammation (Verdecchia et al., 2020).

Initial reports showed mixed evidence of clinical benefit of ACE inhibitors and AT1R blockers (ARBs) in COVID-19, with some showing insignificant effect (Peng et al., 2020; Richardson et al., 2020), as well as reports of protective effect among patients with pre-existing hypertension (Liu Y. et al., 2020; Yang et al., 2020). In a recent meta-analysis of five studies, the odds of death were reduced by a statistically significant 43% among 308 COVID-19 patients using ACE/ARB medications, compared with 1,172 patients not using ACE/ARB medications. A non-significant 19% reduction in the odds of hospitalization among users was also observed (Ghosal et al., 2020). In a separate, larger study of 610 cases and 48,667 high-coverage population–based controls, individuals with hypertension using ARBs were reported to have a 76% lower likelihood of developing COVID-19. However, a similar effect was not reported for ACE inhibitors (Yan et al., 2020).

Apoptosis of alveolar epithelial cells relies on autocrine generation of Ang II, while Ang(1-7) inhibits apoptosis through the Ang(1-7) receptor (Uhal et al., 2011). Exogenous delivery of Ang(1-7) is reported to reduce inflammation and improve lung function in ARDS models (Wosten-van Asperen et al., 2011). Recombinant ACE2 is also reported as a potentially useful therapy in clinical studies of ARDS, producing a rapid decrease in plasma Ang II levels, as well as reduced IL-6 expression (Imai et al., 2007; Zhang and Baker, 2017).

## Pro-Inflammatory Immune Response Initiated by Type-II Alveolar Pneumocytes

The innate pro-inflammatory response to SARS-CoV-2 infection in the lower respiratory tract may be most directly mediated by type-II alveolar pneumocytes, which highly express ACE2. Type-II pneumocytes act as epithelial immune cells and are capable of producing TNF-α, IL-6, IL-1β, MCP-1, and GM-CSF. Infected ACE2+ lung cells, but not uninfected cells, produce high levels of pro-inflammatory cytokines (Wong and Johnson, 2013). The age-related expression profile of ACE2 in uninfected human lung tissue is distinct from that in other ACE2-expressing tissues, showing a positive correlation with immune-cell and interferon-response marker genes in older individuals (>49 years) and a negative correlation in younger individuals (Li et al., 2020).

Local inflammatory cytokine expression in lung tissue of severe CoV infection may differ from that observed in circulating blood. SARS-CoV single-strand RNA is reported to provoke high production of pro-inflammatory TNF-α, IL-6, and IL-12 cytokines *via* activation of TLR7 and TLR8 (both highly expressed in lung tissue), amplifying the innate immune response (Li et al., 2013). Alveolar type-II cells are preferentially infected by SARS-CoV, resulting in the production of pro-inflammatory cytokines, with mRNA encoding IL-6 elevated approximately 10-fold in infected type-II cultures. In contrast, monocytes, monocyte-derived dendritic cells, and alveolar macrophages are not readily infected by SARS-CoV in culture and produce comparatively weak interferon and cytokine levels in response to viral exposure (Qian et al., 2013).

Likewise, the SARS-CoV-2 spike protein is a potent T-cell antigen, and direct activation of COVID-19 patient-derived peripheral blood mononuclear cells (PBMCs) by SARS-CoV-2 peptides in culture results primarily in production of T helper 1 (Th1)–related cytokines. However, IL-6 production is not observed in stimulated PBMCs. This finding suggests that direct antigen-specific T-cell activation may not induce production of IL-6 and that it may instead be mediated by innate immune cells (Weiskopf et al., 2020).

Based on intracellular cytokine staining, peripheral CD14+CD16+ monocytes are also implicated in the production of inflammatory cytokines in COVID-19 (Zhou et al., 2020). However, based on single-cell RNA sequencing of peripheral blood mononuclear cells (PBMCs) from seven COVID-19 cases and six healthy controls, peripheral monocytes and lymphocytes were not found to express substantial amounts of pro-inflammatory cytokines, suggesting that circulating leukocytes do not sufficiently account for COVID-19 cytokine storm (Wilk et al., 2020).

Such expression findings should be interpreted cautiously, as transcripts of many key immune genes demonstrate greater variation and transcription bursts than other genes (Gaublomme et al., 2015). Still, it appears likely that the cytokine storm observed in severe COVID-19 is mediated primarily by type II alveolar cells and local retention of blood cells that have migrated from peripheral circulation to infiltrate lung tissue.

## Induction of Non-Classic Th1 Cells and Intermediate CD14+CD16+ Monocytes

SARS-CoV-2 infection produces rapid activation of pro-inflammatory blood cell lineages. CD4+ Th1 lymphocytes co-expressing IFN*γ* and GM-CSF are reported almost exclusively in ICU patients with COVID-19, with relative absence of these cells in non-ICU patients and healthy controls. The percentage of CD14+CD16+ monocytes is also much greater in ICU patients with severe pulmonary complications. Pathogenic Th1 cells (GM-CSF+IFN*γ*+) are associated with increased proliferation of inflammatory CD14+CD16+ intermediate monocytes expressing both GM-CSF and IL-6. These contribute to the risk of inflammatory cytokine storm (Zhou et al., 2020).

Pathogenic GM-CSF+IFN*γ*+ Th1 cells have been described in inflammatory disease as “non-classic” Th1 cells (or “Th17/Th1” cells) and have been studied in conditions such as multiple sclerosis and juvenile rheumatoid arthritis. These CCR6+ Th17-derived cells have an intermediate gene expression profile between Th1 and Th17, with weaker suppression of Th17-associated genes *RORC2* and *IL-17A* than classic Th1 cells (Mazzoni et al., 2019). Th17 lymphocytes have an unstable phenotype and rapidly shift to a more aggressive non-classic Th1 phenotype in the presence of IL-12 and TNF-α. Inhibitors of TNF-α abrogate this transition (Cosmi et al., 2014). One of the earliest case reports of COVID-19 implicated an increased concentration of CCR6+ Th17 cells as a driver of severe respiratory damage (Xu et al., 2020). The potential therapeutic use of IL-17 inhibitors in COVID-19 has been proposed (Pacha et al., 2020).

The transcription factor Eomes, induced by the combined activity of IL-2 and IL-12, favors the induction of non-classic Th1 cells by selectively suppressing the expression of genes involved in Th17 differentiation. Knockdown of Eomes can be induced by tamoxifen (which also functions as a selective estrogen receptor modulator having tissue-dependent effects as a mixed agonist/antagonist) (Mazzoni et al., 2019). Non-classic Th1 cells are more pathogenic than Th17 cells (Kotake et al., 2017). The preferential induction of these cells is notable, as a comparison of gene expression between severe and non-severe COVID-19 patients reported that, in severe cases, the most significant biological function among differentially expressed genes (DEGs) having downregulated expression was the Th17 cell differentiation pathway (Ouyang et al., 2020).

Intermediate monocytes express the surface molecule CD14 and CD16, which encodes the Fc*γ*III receptor. CD14+CD16+ intermediate monocytes produce high levels of pro-inflammatory TNF-α, coupled with low-to-absent levels of anti-inflammatory IL-10 and have high antigen-presenting capacity. Elevated CD14+CD16+ cells are associated with increased ESR and C-reactive protein (CRP) levels (Ziegler-Heitbrock, 2007). Among monocytes, the highest expression of TNF-α receptor TNFR1 is observed in CD14+CD16+ cells (Hijdra et al., 2013). These monocytes can be mobilized under stress conditions, which may include, but are not dependent on, catecholamine release (Steppich et al., 2000).

Males are reported to have a significantly higher risk of mortality and mechanical ventilation than females in COVID-19, both before and after age-matching (RR, 1.4; 95% CI, 1.2–1.7) (Singh et al., 2020). In this context, it is notable that CD14+ monocytes and monocyte-derived macrophages deprived of 17 beta-estradiol express higher levels of CD16, with significant increases in TNF-α, IL‐1β, and IL‐6 production due to the absence of estrogen (Kramer et al., 2004). Additional factors potentially affecting gender differences in COVID-19 include androgen-mediated transcription of TMPRSS2 and X-linked effects (Wambier and Goren, 2020), as ACE2, androgen receptor, and TLR7 loci are all situated on the X chromosome.

The effects of CD14+CD16+ monocytes in elevating cytokine production and NLR ratios have been studied in other conditions. CD14+CD16+ cells are the preferential targets of Zika virus infection, with amplified proliferation of these cells and a reduction in the percentage and number of classical CD14+CD16- monocytes (Michlmayr et al., 2017). In acute leukemia, CD14+CD16+ monocytes are positively correlated with neutrophil proliferation and negatively correlated with CD4+ lymphocyte count (Jiang et al., 2015). Rheumatoid arthritis is characterized by preferential activation of intermediate CD14+CD16+ monocytes, which contribute to pathogenesis through the production of inflammatory cytokines including TNF-α, IL-1β, and IL-6 (Rana et al., 2018). In patients with type-1 diabetes, CD14+CD16+ monocyte production of IL-1β and IL-6 similarly contribute to pro-inflammatory pathology (Hamouda et al., 2019).

## Skewed Inflammatory Cytokine Production Mediated by Fc and TLR Receptors

Several membrane-bound proteins may contribute to the skewed inflammatory response, elevated cytokine production, and depressed platelet count observed in severe COVID-19. Fc receptors are cell surface proteins that mediate the phagocytosis and cytotoxic destruction of antibody-bound pathogens. Toll-like receptors (TLRs) are pattern-recognition receptors that participate in the innate immune response to extracellular pathogens.

Fc*γ*RIIIA (CD16) expression by monocytes is essential for antibody-dependent cellular toxicity (ADCC), which makes antibody-bound targets, such as virus infected cells, vulnerable to TNF-α–mediated cell death (Yeap et al., 2016). Meanwhile, the monocyte surface molecule CD14 cooperates with TLR2 in response to viral infection, activating nuclear factor-κB (NF-κB)–dependent transcription of genes encoding inflammatory cytokines, which may be inhibited *via* blockade of TLR2-mediated signaling (Zhou et al., 2010). Expression of TLR2 in monocytes is upregulated by IL-6 (Pons et al., 2006). Activation of TLR2 by SARS-CoV spike protein induces the production of inflammatory cytokines, including IL-6, IL-8, and TNF-α (Wang et al., 2007).

In addition to NF-κB activation, CD14-positive monocytes in SARS-CoV patients show an increase in phosphorylated mitogen-activated protein kinase MAPK p38. Augmented p38 MAPK activation in CD14 cells is associated with elevated IL-8 levels (Lee C. H. et al., 2004). The p38 MAPK signaling pathway is also implicated in the death of SARS‐CoV–infected cells (Mizutani, 2007).

Given the observed proliferation of CD14+CD16+ intermediate monocytes in COVID-19 patients with severe pulmonary distress, it is possible that differential activation of Fc*γ* receptor subtypes, particularly Fc*γ*RIIA (inflammatory) and Fc*γ*RIIB (inhibitory), may contribute to an imbalanced inflammatory response. SARS macaque models produce skewed inflammatory cytokine production (including chemoattractants IL-8 and MCP-1) and absence of wound-healing similar to that observed in fatal human cases. Blockade of FcγRIIA reduces these effects (Liu et al., 2019). TNF-α and IL-10 synergistically upregulate FcγRIIA expression, while TNF-α downregulates FcγRIIB expression (Liu et al., 2005). Accordingly, TNF-α inhibition has been suggested as a potential therapeutic in SARS-CoV (Tobinick, 2004). Interestingly, the inhibitory FcγRIIB subtype is selectively upregulated in dendritic cells from RA patients with quiescent disease (Wenink et al., 2009).

Blockade of FcR activation *via* IVIG has been suggested for severe pulmonary inflammation and lung injury in SARS-CoV-2 (Fu et al., 2020). The anti-inflammatory effect is associated with its ability to recruit surface expression of the inhibitory Fc receptor FcγRIIB (Samuelsson et al., 2001). Among potentially repurposed therapeutics, IVIG is not without dangers (renal failure, thrombosis), and effectiveness is not established in MERS (Mustafa et al., 2018). Alternatively, human polyclonal immunoglobulin G from bovines has been reported to inhibit MERS-CoV *in vivo* (Luke et al., 2016).

Because depressed platelet count and dysregulated immune function is observed in COVID-19, the mediating role of Fc*γ* receptors in immune thrombocytopenia (ITP) may also be informative. In ITP, loss of self-tolerance to platelet protein leads to destruction of platelets and precursor megakaryocytes by binding of platelets to Fc receptors on macrophages. The inhibitory Fc*γ*RIIB receptor subtype (FCGR2B) prevents consumption by macrophages. Exogenous soluble Fc*γ*RIIB competitively binds antibody-bound platelets (Luke et al., 2016) and prevents autoantibody production (Shih et al., 2014). In contrast, Fc*γ*RIIA (FCGR2A) significantly aggravates the severity of antibody-mediated thrombocytopenia (McKenzie et al., 1999). Blocking Fc*γ*RIIIA (CD16) has also been shown to reduce ITP in mouse models (Flaherty et al., 2012).

In addition to viral entry *via* ACE2, antibodies against coronavirus spike proteins (anti-spike-S-IgG) can induce antibody-dependent enhancement (ADE) of viral entry *via* type II Fc*γ* receptors. Such enhancement has been studied in SARS-CoV infection (Wang et al., 2014) and appears to be dependent on the activation of Fcγ receptor II. Among FcR subtypes, FcγRIIA (CD32A) appears to mediate infectivity most efficiently (Jaume et al., 2011). In MERS-CoV, neutralizing antibodies can bind to the spike protein and enable alternative entry into FcγRIIA expressing cells (Wan et al., 2020). Accordingly, care in the selection of antigens is essential in the design of vaccine and antibody-based therapeutic strategies in order to avoid the potential for ADE.

Risk-genotypes associated with severe inflammatory pathology may be informative in the context of COVID-19. The FcγRIIA-R/R131 (rs1801274) genotype induces variation in the Fc*γ*RIIA receptor, while the CD14-159CC (rs2569190) genotype induces variation in CD14-mediated pro-inflammatory cytokine induction. Both are risk-genotypes for severe SARS (Yuan et al., 2005; Yuan et al., 2007) as well as aberrant immune response in pneumonia (Yuan et al., 2005), myasthenia gravis (van der Pol et al., 2003; Aricha et al., 2011), and acute asthma (Martin et al., 2006; Zhou et al., 2019).

## Neutrophil Induction and Lung Infiltration

Severe SARS-CoV-2 infection is characterized by high neutrophil infiltration into lung tissue. In a study of 222 COVID-19 patients, disease severity was associated with significantly higher levels of both anti-virus IgG (IgG) and NLR ratio. Severity rates for patients with NLR^high^IgG^high^, NLR^high^IgG^low^, NLR^low^IgG^high^, and NLR^low^IgG^low^ phenotypes were 72.3, 48.5, 33.3, and 15.6%, respectively (p < 0.0001). Recovery rates for severe patients with these phenotypes were 58.8, 68.8, 80.0, and 100%, respectively (p = 0.0592). Notably, high NLR patients expressed the highest levels of IL-2, IL-6, and IL-10, with fatalities observed only in these patients (Zhang et al., 2020b).

Neutrophils comprise the majority of infiltrating cells into tissues undergoing inflammation. Transcriptional analysis of genes induced by SARS-CoV-2 features a host response characterized by weak induction of type I and type III interferons, coupled with enrichment of genes associated with cell death, leukocyte activation, and chemokine recruitment, including IL-1A, MCP-1 (CCL2), and IL-8 (CXCL8) (Blanco-Melo et al., 2020). In ARDS, MCP-1 and IL-8 induce chemotaxis of pro-inflammatory neutrophils into the lungs, where they are retained in the capillary bed and migrate into the alveolar space, contributing to cytokine production, formation of microthrombi, and cell death. GM-CSF, IL-8, and IL-2 contribute to delayed apoptosis, prolonging the amplified inflammatory response. In animal models of neutrophil-driven lung injury, cyclin-dependent kinase (CDK) inhibitors are reported to reduce inflammation and improve resolution by inducing neutrophil apoptosis (Potey et al., 2019). CDK9 is specifically implicated in this process (Wang et al., 2012).

Neutrophils can target pathogens and create a physical barrier to their migration by releasing NETs comprised of mesh-like extracellular DNA. NETs are observed at high levels in COVID-19 patients. Patient sera induce healthy control neutrophils to undergo NETosis. However, NETs may contribute to cytokine release and progression to respiratory failure (Zuo et al., 2020) and contribute to thrombosis *via* platelet-neutrophil interaction (Laridan et al., 2017).

## Adhesion and Tissue Retention of Inflammatory Leukocytes

The pathological inflammatory response observed in COVID-19 may be mediated by adhesion of hyperactivated and aggressive T-cells, monocytes, and neutrophils retained from peripheral circulation by vascular endothelia. Endothelial barrier degradation, capillary leakage, and extravasation into inflamed tissue may then contribute to the DAD observed in severe cases.

Phenotypic profiling of circulating leukocytes in critical COVID-19 patients indicates high activation of S-protein specific T-cells producing inflammatory cytokines, coupled with depletion of CD4+ and CD8+ T-cells expressing the LFA-1 integrin subunit CD11a. Conversely, recovery from respiratory distress is accompanied by a reversal of CD11a+ cell depletion (Anft et al., 2020). Hyperactivated T-lymphocytes and inflammatory macrophages recruited by chemokine signaling to lung tissue exhibit strong interaction with epithelial cells, contributing to increased cell death and lung injury. Elevated markers of immune cell trafficking in COVID-19 include MCP-1 and LFA-1. As monocyte recruitment and epithelial damage can be induced by binding of MCP-1 to ligands CCR1 or CCR5, blockade of these ligands has been suggested as a potential therapeutic approach (Chua et al., 2020).

Adhesion of inflammatory CD14+CD16+ monocytes and neutrophils to vascular endothelia is mediated by interaction of LFA-1 with its ligand, intercellular adhesion molecule ICAM-1. Inflammatory cytokines IL-1 and TNF-α induce ICAM-1 expression on endothelial cells. Expression of ICAM-1 selectively enhances adhesion of inflammatory non-classical and intermediate CD16+ monocytes under flow, with no effect on CD16- monocytes (Regal-McDonald et al., 2019). Docosahexaenoic acid (DHA) is reported to inhibit TNF-α-induced ICAM-1 expression (Lin H. C. et al., 2019), with similar inhibition of ICAM-1 expression reported for eicosapentaenoic acid (EPA) in aortic endothelia (Huang et al., 2015).

ICAM-1 facilitates cytokine-induced adhesion of neutrophils to vascular endothelia (Tonnesen, 1989). Notably, upregulation of ICAM-1 expression and inflammatory leukocyte recruitment is observed in ARDS (Müller et al., 2002) and respiratory syncytial virus (RSV) disease (Arnold and König, 2005). Similar upregulation is observed in Ang II-induced macrophage infiltration and cardiovascular pathology, which is ameliorated by ICAM-1 blockade (Lin Q. Y. et al., 2019). Blockade of ICAM-1 is also reported to markedly reduce pulmonary barrier damage in ARDS (Svedova et al., 2017).

Extravasation of CD14+CD16+ intermediate monocytes is mediated by secretion of MMP-9, a protease that degrades extracellular matrix proteins, resulting in the release of matrix-bound VEGF-A and increased vascular membrane permeability (Sidibe et al., 2018), In COVID-19 patients with respiratory failure, a significant increase is observed in circulating MMP-9, strongly correlated with neutrophil count (Ueland et al., 2020).

COVID-19 respiratory failure thus features co-expression of inflammatory cytokines with regulators of leukocyte recruitment and vascular integrity. This suggests a mechanism by which inflammatory leukocytes may degrade the alveolar-capillary barrier, with resulting destruction of lung tissue. Notably, electron microscopy of post-mortem lung tissue reveals extensive opening of junctional complexes. Hyperalbuminemia in severe COVID-19 patients, consistent with vascular permeability and capillary leakage, is strongly associated with mortality (Wu M. A. et al., 2020).

The potential importance of this mechanism in COVID-19 pathology is underscored by transcriptional and proteomic profiling. In bronchial epithelial cells infected with SARS-CoV-2, DEGs are enriched for members of pathways related to NF-κB, TNF-α, and IL-17 signaling. Specific genes shared by these pathways include *MMP9*, *ICAM1*, *CSF3*, and *IL6* (Enes and Pir, 2020). A protein-protein interaction network of DEGs shared between COVID-19, MERS, SARS, H1N1, and Ebola identifies *ICAM1*, *VEGFA*, *MMP9*, *IL6*, *TNF*, *IL-8*, *IL1B*, *STAT1*, *TLR2*, *TLR1*, *IRF7*, and *CXCL1* as hub genes (Alsamman and Zayed, 2020). Proteomic profiling of blood samples from COVID-19 patients identifies ICAM-1 and FCGR3A (CD16) as the most significant proteins in the classification of short vs. extended disease course (Tang). Likewise, in post-mortem lung tissue, IL-6, TNF-α, ICAM-1, and CASP-1 (an activator of inflammatory response and cell death) show significantly higher tissue expression, compared with control and H1N1 samples (Nagashima et al., 2020).

Although SARS-CoV-2 infection in pediatric cases is generally associated with asymptomatic resolution, a perplexing minority of children present with Kawasaki disease (KD)–like features, alternatively described as multisystem inflammatory syndrome (MIS). These patients present with high inflammatory markers, early gastrointestinal symptoms, and acute myocarditis, with therapeutic immune globulin reportedly contributing to recovery (Toubiana et al., 2020; Belhadjer et al., 2020). These cases may potentially be understood in the context of the same mechanisms of inflammatory leukocyte infiltration implicated above.

Specifically, acute KD is associated with increased proliferation of CD14+CD16+ intermediate monocytes (Katayama et al., 2000), while diminished inflammation in response to plasma exchange therapy is associated with a significant reduction in the percentage of CD14+CD16+ intermediate monocytes, relative to total leukocytes (Koizumi et al., 2019). The acute phase of KD also features transient depletion of CD11a-expressing T-cells from peripheral blood (Furukawa et al., 1993). In cultured vascular endothelial cells, patient sera from acute phase KD induces significantly higher expression of ICAM-1 than quiescent sera, with TNF-α contributing to ICAM-1 expression (Inoue et al., 2001). In KD cases exhibiting coronary artery abnormalities, a high and unresponsive NLR ratio is associated with resistance to IVIG treatment (Cho et al., 2017). Thus, the KD-like symptoms observed in a subset of pediatric COVID-19 cases are broadly consistent with the inflammatory mechanisms described in the proposed pathway.

## Weak Interferon Defense and Neutrophil-Driven Cytotoxicity in Lung Epithelia

SARS-CoV-2 infection is associated with increased levels of pro-inflammatory cytokines (Chen et al., 2020; Zhang, Guo, et al., 2020), yet the immune response in lung tissue features a relatively impaired response of type I (α/β), II (*γ*), and III (λ) interferons (Chu et al., 2020), along with down-regulation of interferon-induced genes. This contrasts with the interferon response in SARS-CoV, where preferential infection of alveolar type-II cells results in a marked increase of IFN-β and IFN-λ (IL-29) production (Qian et al., 2013).

The suppressed IFN-λ response observed in COVID-19 may be a key factor mediating viral infectivity. In human lung tissues, SARS-CoV-2 demonstrates markedly higher infectivity and replication than that of SARS-CoV, generating 3.2 times the number of infectious virus particles within 48 hours of infection (Chu et al., 2020).

While IFN-α and IFN-β receptors are primarily expressed on peripheral blood cells, IFN-λ receptors have restricted expression, preferentially defending epithelial cells, including respiratory pneumocytes. IFN-λ expression thus provides an initial line of defense to restrict viral replication in the upper airways, suppress excessive inflammation of the lower airways, and maintain the integrity of cellular barriers to inflammatory injury (O’Brien et al., 2020; Broggi et al., 2020).

In Dengue infection, IFN-λ inhibits replication of the DENV-2 virus in a dose-dependent manner *in vitro* (Palma-Ocampo et al., 2015). The rs7247086 variant of *IFNL1* (the T allele) is reported to be protective against DHF, suggesting that *IFNL1* may play a role in the pathogenesis and elevated cytokine expression observed in this condition (Arayasongsak et al., 2020).

Notably, MERS-CoV encodes two accessory proteins, NS4a and NS4b that contribute to suppression or evasion of innate antiviral immune pathways. In particular, both deletion of NS4a and mutation of catalytic or nuclear localization sites of NS4b result in increased expression of IFN-λ1 (Comar et al., 2019). The weak interferon response observed in COVID-19 suggests that the possibility that one or more SARS-CoV-2 viral proteins may exert a similar effect in suppressing IFN-λ expression, weakening front-line innate immune defense against viral infectivity. Similarly, viral proteins of RSV, the most important respiratory virus among infants, antagonize IFN-mediated epithelial protection. Exogenous IFN-λ1 confers prophylactic benefit against viral infection (Villenave et al., 2015).

A recent genome-wide association study examined 300,000 loci to identify genetic factors associated with ACE2 expression in the presence of RNA virus infection. The most significant association was identified in three SNPs within the IFN-λ region of chromosome 19, controlling expression of IFNL3 and IFNL4. In the presence of RNA virus infection, ACE2 expression shows a significant negative correlation with IFN pathway genes. One of these SNPs is located near a frameshift mutation that disables the production of IFN-λ4 (Ansari et al., 2020). As both ACE2 and receptors for IFN-λ are preferentially expressed on type II alveolar pneumocytes, their association may be relevant in COVID-19 pathology, as suppressed IFN-λ expression coupled with elevated ACE2 expression could simultaneously suppress epithelial defense while amplifying the viral load.

Weak induction of IFN-λ in COVID-19 may be an important amplifier of cytokine production by impairing the control of inflammatory neutrophil responses. In animal models of ARDS induced by influenza-A virus (IAV) infection, neutrophils comprise the majority of infiltrating cells and are the primary source of pro-inflammatory cytokines. Neutrophils also express high levels of the interferon-lambda receptor IFNLR1 in proximity to epithelial cells, allowing IFN-λ to mediate sustained local anti-viral defense without amplifying inflammation. Accordingly, exogenous administration of pegylated recombinant IFN-λ in IAV-induced ARDS suppresses viral replication and improves lung function (Galani et al., 2017). IFN-λ also suppresses the migration of neutrophils and their proclivity to NETosis, thereby enabling the suppression of thromboinflammation (Chrysanthopoulou et al., 2017).

Low levels of IFN-λ in COVID-19 also appear likely to skew immune response toward neutrophil proliferation and suppressed lymphocyte response, contributing to the thrombosis, pro-inflammatory cytokine production, and fatality observed among NLR^high^ patients. Exogenous IFN-λ may reduce these consequences. CD14+ monocytes quickly express the IFN-λ receptor IFNLR1 upon differentiation to macrophages. IFN-λ stimulates the cytotoxic and phagocytic capacity of macrophages, as well as the secretion of cytokines that mediate T and NK-cell migration and cytotoxicity (Read et al., 2019).

## Cytokine Storm Featuring High Expression of IL-6 and TNF-α

Increased IL-6 is an early indicator of cytokine release syndrome in COVID-19 patients (Wang et al., 2020). IL-6 concentrations are increased 2.9-fold in patients with complicated COVID-19 vs. uncomplicated (Coomes and Haghbayan, 2020), and IL-6 levels are predictive of respiratory failure (Herold et al., 2020; Zhang et al., 2020a).

The SARS-CoV spike protein induces (TNF-α converting enzyme) TACE-dependent shedding of the extracellular ACE2 receptor domain, resulting in loss of ACE2 function and production of TNF-α. NL63-S, a common cold coronavirus serotype, also uses ACE2 for entry, but does not induce similar ACE2 shedding or TNF-α production (Haga et al., 2008). TACE antagonists have been suggested as an approach to inhibit TNF-α and attenuate disease severity in SARS-CoV (Tobinick, 2004).

Cytokine storm on the IL-6/TNF-α axis appears likely to be mediated by phosphorylation of the NF-κB subunit p65. In SARS-CoV infection, the viral spike protein induces activation of NF-κB *via* IkB-α degradation, resulting in production of IL-6 and TNF-α (Wang et al., 2007). The viral nucleocapsid protein of SARS-CoV can also bind the NF-κB regulatory element on the IL-6 promoter, and activity is highest when the p65 subunit is present (Zhang et al., 2007).

Regulatory elements in the ACE2 gene control the transcription of PIR (pirin), a negative regulator of NF-κB subunit RELA (p65). SARS-CoV-2 disruption of ACE2 is proposed to reduce PIR expression (Fadason et al., 2020). PIR is proposed to function as a reversible switch that enables NF-κB response to changes in redox levels (oxidative stress) in the cell nucleus (Liu et al., 2013). Repression of PIR ablates inhibition of IL-6 expression (Wu et al., 2017).

Inhibition of NF-κB activation has been suggested as a therapeutic strategy to increase survival in SARS-CoV infection (DeDiego et al., 2014). Inhibition of JAK signaling may block p65 phosphorylation and attenuate proinflammatory cascade (Yang et al., 2017). Tocilizumab, a well-tolerated blocker of the IL-6 receptor, may have potential to dampen cytokine release syndrome in COVID-19 (Zhang C. et al., 2020). Because catecholamines augment the production of IL-6 and other inflammatory cytokines, α-1 adrenergic receptor inhibition (e.g., prazosin) has also been suggested as a candidate that may provide prophylactic benefit against cytokine storm (Konig et al., 2020).

Use of low molecular weight heparin is reported to be associated with improvement in aberrant coagulation and a reduction of IL-6 levels (Shi et al., 2020), and is reported to increase survival in COVID-19 (Negri et al., 2020; Tang et al., 2020). However, elevated anti-heparin-PF4 antibodies have been observed in severe COVID-19 patients, even in the absence of heparin exposure, and may contribute to heparin-induced thrombocytopenia, *via* binding of antibody-heparin complexes to the platelet FcγRIIA receptor (Liu X. et al., 2020). For that reason, the use of alternative anticoagulants (other than coumadin, which may provoke thrombotic complications) may be indicated (Izak and Bussel, 2014).

## Discussion

The rapid case growth and high fatality rate of COVID-19 have posed an urgent global health challenge. Major uncertainties exist in ascertainment, and case reports are likely to exclude large numbers of subclinical or asymptomatic cases that may contribute to infectivity and confound containment efforts. Meanwhile, conditional on cases that have been reported and confirmed, the global case fatality rate of the disease exceeds 4.8%, with the United States experiencing the highest number of fatalities (127,000) through June 2020 (ncov-CSSE, 2020).

Despite incomplete knowledge of the pathophysiology relating to the novel coronavirus SARS-CoV-2, the proliferation of initial reports and small-scale studies carry stronger information content than may be evident amid the “noise” of this emerging literature, when integrated in the context of prior research on other CoV serotypes, ARDS, and related inflammatory conditions. From a noise-reduction perspective, information content can often be amplified by extracting jointly correlated signals from what might otherwise be individually weak sensors. The tractable pathway presented here is reflective of that effort.

**Figure 1:**
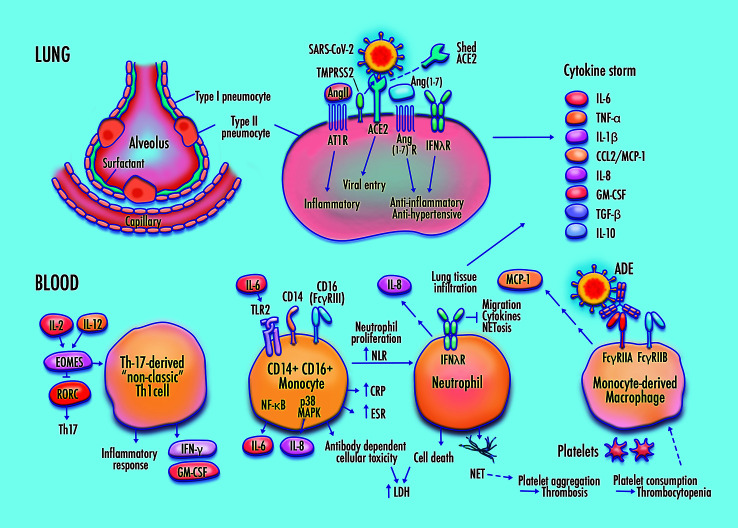
Proposed features of cellular and molecular pathophysiology in COVID-19. Membrane fusion and cytoplasmic entry of SARS-CoV-2 virus *via* ACE2 and TMPRSS2-expressing respiratory epithelial cells, including pulmonary type-II pneumocytes, provokes an initial immune response featuring inflammatory cytokine production coupled with a weak interferon response, particularly in IFN-λ–dependent epithelial defense. Differentiation of non-classic pathogenic T-cells and pro-inflammatory intermediate monocytes contributes to a skewed inflammatory profile, mediated by membrane-bound immune receptor subtypes (e.g., Fc*γ*RIIA) and downstream signaling pathways (e.g., NF-κB p65 and p38 MAPK), followed by chemotactic infiltration of monocyte-derived macrophages and neutrophils into lung tissue. Endothelial barrier degradation and capillary leakage contribute to alveolar cell damage. Inflammatory cytokine release, delayed neutrophil apoptosis, and NETosis contribute to pulmonary thrombosis and cytokine storm. These mechanisms are concordant with observed clinical markers in COVID-19, including high expression of inflammatory cytokines on the TNF-α/IL-6 axis, elevated neutrophil-to-lymphocyte ratio (NLR), DAD *via* cell apoptosis in respiratory epithelia and vascular endothelia, elevated lactate dehydrogenase (LDH), erythrocyte sedimentation rate (ESR), and CRP, high production of neutrophil extracellular traps (NETs), depressed platelet count, and thrombosis.

Part of this analysis, by necessity, includes findings from early reports and pre-published data that may be modified or contradicted by subsequent studies. Accordingly, some elements of this pathway may require revision as new findings emerge. **Figure 1** illustrates this pathway.

Among the benefits of a coherent biological pathway, consistent with the observed clinical course of SARS-CoV-2, is that it connotes multiple points of intervention for potential therapeutic candidates. Emphatically, the candidates described below are not prescriptive but are instead discussed here to provoke pathway-informed investigation.

Potential investigational therapeutics consistent with the proposed COVID-19 pathway are listed in **Table 1**. Specific candidates are indicated as examples and do not comprise an exhaustive list. These candidates are not prescriptive but are instead intended to provoke further research and pathway-informed investigation.

**Table 1:** Potential investigational therapeutics consistent with proposed COVID-19 pathway.

**Therapeutic candidate (not exhaustive)**	**Class**	**Potential mechanism and basis for investigation**
Losartan, Irbesartan	Angiotensin II receptor AT1R blocker (ARB)	Blockade of pro-inflammatory, pro-hypertensive Ang II effects
Recombinant ACE2, Ang (1-7)	Exogenous RAS modulators	Restoration of anti-inflammatory, anti-hypertensive Ang(1-7) effect
Prazosin	Alpha-adrenergic blocker	Reduction of catecholamine-related amplification of cytokine response
Pasireotide	Somatostatin analogue	Reduction of cortisol-mediated NLR
Pegylated IFN-λ	Interferon-III	Augmented defense of respiratory epithelium, reduced cytokine production, NETosis and thrombosis
Calcitriol, Melatonin	Natural hormone supplement	Prophylaxis, reduced cytokine induction
Lopinavir, Camostat	Protease inhibitor	Disruption of viral entry
Remdesivir	Antiviral agent	Reduction of viral replication
Chlorpromazine, Triflupromazine	Dopamine D2 receptor antagonist	Reduction of viral titer via disruption of clathrin-mediated endocytosis
Emetine, Ivermectin,Hydroxychloroquine	Anti-parasitic	Prophylactic reduction of viral titer
Imatinib, Dasatinib	Abelson (ABL) kinase inhibitor	Blockade of host-virus membrane fusion
Toremifene, Tamoxifen	Estrogen receptor modulator (tissue-dependent mixed agonist/antagonist)	Antiviral activity and inhibition of non-classic Th1 induction, potentially via receptor-independent mechanisms
Estradiol	Steroid hormone	Inhibition of CD16 and proliferation of inflammatory intermediate monocytes
DHA, EPA	n-3 polyunsaturated fatty acid	Reduced ICAM-1-mediated leukocyte adhesion and inflammatory response
Doxycycline	Tetracycline antibiotic	Antibiotic, anti-inflammatory effect on cytokine expression and MMP activity
Dexamethasone, Methylprednisolone	Glucocorticoid	Reduced inflammatory response
Sekukinumab, Broadalumab	IL-17 inhibitor	Reduced inflammatory response
Tocilizumab, Siltuximab	IL-6 inhibitor	Reduced inflammatory response
Etanercept	TNF inhibitor	Reduced inflammatory response
Tofactinib, Fedratinib	JAK inhibitor	Inhibition of NF-κB p65 signaling
Alvocidib	Cyclin-dependent kinase (CDK) inhibitor	Reduced inflammatory response and neutrophil-mediated cell death
Fc*γ*RIIB	Exogenous Fc receptor delivery	Reduced inflammatory response, potential inhibition of platelet consumption

Initial interventions with potential benefit early in SARS-CoV-2 infection may include approaches focused on augmenting epithelial defense, reducing viral load, and modifying inflammatory signaling. Potential candidates include the use ACE inhibitors and AT1R blockers (ARBs) to reduce the hypertensive and pro-inflammatory effects of Ang II, exogenous Ang(1-7), recombinant ACE2, pegylated IFN-λ, early administration of IFN-I, and α-1 adrenergic receptor inhibition.

In a study of 77 COVID-19 patients, treatment with IFN-α2b significantly reduced the duration of detectable virus in the upper respiratory tract, and reduced the duration of elevated IL-6 and CRP levels (Zhou Q. et al., 2020). However, evidence from SARS and MERS cases suggests that while early delivery of IFN-I can reduce viral replication, later delivery may amplify risk by elevating pro-inflammatory response (Channappanavar et al., 2016; Channappanavar et al., 2019).

Among conservative, well-tolerated therapeutic candidates, melatonin exerts a protective effect on vascular endothelia by inhibiting NF-κB induced expression of MMP-9 (Qin et al., 2012). It is also reported to protect lung tissue from hypoxic stress by downregulating TNF, IL-6, and VEGF expression, with quercetin providing additional prophylactic effect (Al-Rasheed et al., 2017). Vitamin D attenuates TLR-mediated induction of inflammatory cytokines (Thota et al., 2013). This mechanism may be relevant in COVID-19 as low plasma levels of vitamin D are reported in SARS-CoV-2 infected individuals and significantly contribute to the risk of infection and hospitalization (Merzon et al., 2020). Calcitriol, the active form of vitamin D, is also reported to directly reduce the virus-induced cytopathic effect of SARS-CoV-2 infection in cultured human respiratory epithelial cells (Mok et al., 2020). The combination of melatonin and vitamin D has been proposed as a potentially synergistic intervention in COVID-19 (Martín Giménez et al., 2020).

Several classes of therapeutics may have benefit as potential viral entry inhibitors. In a screening of 290 compounds for antiviral activity against SARS-CoV and MERS-CoV, those promoting at least 50% viral inhibition in Vero E6 cells *in vitro* with little or no toxicity included selective estrogen receptor modulators (SERMs) (e.g., toremifene and tamoxifen), Abelson kinase (ABL) inhibitors (e.g., imatinib and dasatinib), dopamine D2 receptor antagonists (e.g., chlorpromazine and triflupromazine), and antiparasitic agents (e.g., hydroxychloroquine and emetine) (Dyall et al., 2014). Research involving additional cell lines may be informative in this context, because while SARS-CoV-2 can be isolated from Vero E6 cells, cells engineered to express TMPRSS2 display a nearly 10-fold increase in SARS-CoV-2-infected cells than parental Vero E6 cells (Matsuyama et al., 2020).

SERMs such as toremifene are reported to potently inhibit Ebola virus, even without detectable expression of estrogen receptors, suggesting that SERMs may affect viral activity through an alternative pathway (Johansen et al., 2013). In CD14+ monocytes, SERMs are reported to reduce inflammatory signaling by downregulating TNF-α–stimulated NF-κB activation and to promote macrophage differentiation toward an M2 anti-inflammatory/repair phenotype (Polari et al., 2018). Toremifene was among two network-predicted therapeutics, along with the AT1R blocker irbesartan, with the strongest correlation between CoV-induced transcriptomes and drug-induced transcriptomes and having literature-based antiviral evidence (Zhou Y. et al., 2020).

ABL inhibitors are reported to have potent effect against SARS-CoV and MERS-CoV cell fusion, which is required for cytoplasmic delivery of the viral genome (Coleman et al., 2016). The D2 receptor antagonist chlorpromazine is reported to inhibit clathrin-mediated endocytosis in both SARS-CoV (Inoue et al., 2007) and MERS-CoV (Liang et al., 2018).

Several antiparasitic agents are recognized for exhibiting antimicrobial and anti-inflammatory properties, suggesting potential benefit against SARS-CoV-2 infection. For example, ivermectin interferes with the nuclear import of proteins encoded by several RNA viruses and is reported to exert anti-viral action against SARS-CoV-2 in Vero cells (Caly et al., 2020). Early evidence suggests that ivermectin treatment may be associated with reduced mortality risk in patients with COVID-19, particularly in those requiring oxygen support or mechanical ventilation (Rajter et al., 2020).

Hydroxychloroquine has been broadly used during the SARS-CoV-2 epidemic, with evidence of potential prophylactic effect (Colson et al., 2020) mediated by reduced viral replication (Keyaerts et al., 2004) and interference with ACE2 binding (Vincent et al., 2005). Chloroquine is also reported to reduce secretion of IFN-γ and IL-17 in activated Th1 and Th17 cells, respectively (Schmidt et al., 2017). However, evidence of therapeutic benefit for hospitalized patients has not been clearly established (Magagnoli et al., 2020; Shamshirian et al., 2020). In addition to potential risks of retinopathy and arrhythmia, combination therapy with azithromycin is reported to be associated with increased risk of heart failure and cardiovascular mortality (Lane et al., 2020).

A randomized, controlled trial of remdesivir including more than 1000 patients reported a reduction in average time to recovery to 11 days for the treatment group vs. 15 days for patients assigned to placebo. A small but insignificant reduction in the risk of fatality was also observed among treated patients (Ledford, 2020). In a screening of 16 therapeutic candidates specifically targeting SARS-CoV-2, the antiparasitic agent emetine was reported among four compounds achieving at least 50% in-vitro inhibition, along with remdesivir, lopinavir, and homorringtonine. Synergy between remdesivir and emetine was observed, enabling reduced dosages to achieve significant reduction in viral yield (Choy et al., 2020). In the context of SARS-CoV-2, adjuvant use of emetine may be of particular interest, given that emetine has a well-established role in enhancing interferon activity (Schellekens et al., 1975) and is reported to disrupt viral entry and replication (Yang et al., 2018). Considerations include pregnancy and cardiovascular risk.

The broad spectrum antibiotic doxycycline has been shown to exert anti-inflammatory effects by interfering with the expression of IL-6, IL-8, and TNF-α, reducing the recruitment of neutrophils and lymphocytes into inflamed tissue, and suppressing the activity of metalloproteinases (MMPs) (Di Caprio et al., 2015). Notably, doxycycline treatment was reported to reduce mortality by half in human patients with DHF, with survival associated with significant reductions in TNF and IL-6 levels (Fredeking et al., 2015). Administration of doxycycline also significantly decreases MMP-mediated capillary leakage and alveolar damage in virus-infected mice (Ng et al., 2012). These properties suggest potential therapeutic benefit of doxycycline across multiple fronts of COVID-19 immunopathology.

Corticosteroids are commonly used in the treatment of inflammatory conditions, but timing and duration of use are important considerations in the context of COVID-19. In SARS, early corticosteroid treatment (<7 days of illness) was associated with an increase in subsequent viral load (Lee N. et al., 2004). However, the use of steroids may be beneficial at the point of disease progression to acute respiratory distress and cytokine storm (Tomazini et al., 2020). Methylprednisolone use is reported to reduce the risk of death in patients with COVID-19 pneumonia that has progressed to ARDS (Wu C. et al., 2020). This result is consistent with clinical evidence in SARS, where pulse methylprednisolone was reported to be beneficial in a subset of patients with critical illness. Prolonged steroid administration without effective antimicrobial support is discouraged due to the risk of secondary infection (Tai, 2007).

In a randomized controlled trial comparing 2104 COVID-19 patients receiving dexamethasone and 4321 patients receiving standard-of-care, dexamethasone treatment reduced the risk of death by one-third in patients requiring invasive mechanical ventilation and by one-fifth in patients requiring oxygen without invasive ventilation. Dexamethasone did not reduce mortality risk in patients that had not progressed to the need for respiratory support at the time of randomization (Horby et al., 2020). However, in non-intubated patients with COVID-19 pneumonia, combination therapy including corticosteroids and tocilizumab is reported to increase survival (Mikulska et al., 2020).

Steroid use has been suggested as a possible factor contributing to the elevated NLR ratio observed in SARS patients. However high NLR is observed even in steroid-naïve patients, and elevated serum cortisol is reported to be correlated with the degree of neutrophilia and lymphopenia (Panesar et al., 2004). High adrenocorticotropic hormone (ACTH) production and induced cortisol release in response to SARS-CoV infection has been suggested to mimic the effect of corticosteroids in driving T-lymphocytes out of peripheral circulation (Panesar, 2003). The somatostatin analogue pasireotide may attenuate the skewed neutrophil/lymphocyte response observed in COVID-19.

Additional pathway-informed candidate therapeutics targeting molecular mediators of the COVID-19 hyperinflammatory response include biologics such as TNF-α inhibitors, IL-6 inhibitors, tamoxifen-mediated inhibition of Eomes, IL-17 inhibitors, CDK inhibition, exogenous delivery of soluble Fc*γ*RIIB, and JAK inhibitors. Among TNF inhibitors, etanercept was proposed as a potential first-line choice in SARS-CoV based on considerations of safety, short-half life, and limited immunogenicity (Tobinick, 2004). Early evidence relating to compassionate use of IL-6 inhibitors in SARS-CoV-2 (tocilizumab and siltuximab) appears promising, with unfavorable outcomes generally associated with treatment-resistant increases in IL-6. Well-designed clinical trials appear justified (Khan et al., 2020).

The high infectivity, rapid case growth, and severe outcomes of the SARS-CoV-2 epidemic have created an urgent global health crisis and a pressing need for therapeutic approaches to contain the number of fatalities. This epidemic has emerged in the context of a rich existing literature detailing aspects of cellular and molecular pathways affected by prior CoV serotypes and related conditions. Much of the emerging literature specific to SARS-CoV-2 is strongly consistent with these findings, and also features informative differences, particularly in lung tissue (e.g., weaker interferon response, suppressed epithelial defense, and elevated pulmonary infectivity).

The resulting synthesis enables construction of a coherent biological pathway that suggests multiple points of investigation for potential therapeutic candidates. Given the high case fatality rate of COVID-19, such candidates may help to bridge an urgent gap. While results from ongoing randomized controlled clinical trials remain essential, critical patients may benefit in the interim from the estimation of preliminary odds ratios relating to repurposed therapeutics, based on outcomes of COVID-19 patients having existing exposure to pathway-relevant candidates.

## Author Contributions

The author confirms being the sole contributor of this work and has approved it for publication.

## Conflict of Interest

The author declares that the research was conducted in the absence of any commercial or financial relationships that could be construed as a potential conflict of interest.
